# Clinical Utility of the FilmArray^®^ Blood Culture Identification (BCID) Panel for the Diagnosis of Neonatal Sepsis

**DOI:** 10.3390/microorganisms11030732

**Published:** 2023-03-12

**Authors:** María Caunedo-Jiménez, Belén Fernández-Colomer, Jonathan Fernández-Suárez, Rosa Patricia Arias-Llorente, Sonia Lareu-Vidal, Laura Mantecón-Fernández, Gonzalo Solís-Sánchez, Marta Suárez-Rodríguez

**Affiliations:** 1 Division of Neonatology, Department of Pediatrics, Central University Hospital of Asturias, Av. Roma s/n, E-33011 Oviedo, Spain; 2 Department of Microbiology, Central University Hospital of Asturias and Instituto de Investigación Sanitaria del Principado de Asturias, E-33011 Oviedo, Spain

**Keywords:** neonatal sepsis, blood culture, early-onset sepsis, late-onset sepsis, FilmArray^®^ blood culture identification panel, neonates

## Abstract

This prospective single-center study was designed to assess the clinical utility of the FilmArray^®^ blood culture identification (BCID) panel for improving the diagnostic accuracy in neonatal sepsis. Results obtained using the FilmArray^®^ BCID panel were correlated with results of blood culture in all consecutive neonates with suspicion of early-onset (EOS) and late-onset sepsis (LOS) attended in our service over a two-year period. A total of 102 blood cultures from 92 neonates were included, 69 (67.5%) in cases of EOS and 33 (32.3%) in LOS. The FilmArray^®^ BCID panel was performed in negative culture bottles at a median of 10 h of blood culture incubation (IQR 8–20), without differences by the type of sepsis. The FilmArray^®^ BCID panel showed a 66.7% sensitivity, 100% specificity, 100% positive predictive value, and 95.7% negative predictive value. There were four false-negative cases, three of which were *Streptococcus epidermidis* in neonates with LOS, and there was one case of *Granulicatella adiacens* in one neonate with EOS. We conclude that the use of the FilmArray^®^ BCID panel in negative blood cultures from neonates with clinical suspicion of sepsis is useful in decision-making of starting or early withdrawal of empirical antimicrobials because of the high specificity and negative predictive values of this assay.

## 1. Introduction

Neonatal sepsis remains a leading cause of morbidity and mortality both among preterm and term neonates [1]. Sepsis in newborns is a serious medical condition, and when neonatal sepsis is clinically suspected, a blood sample for culture should be obtained, and empirical antimicrobial treatment should be initiated since delayed treatment is commonly associated with adverse outcomes [2,3]. Antimicrobials are usually maintained for 48 h, which is the standard time needed for identification of most microorganisms, including slowly growing pathogens. However, antimicrobial exposure, particularly during the first week of life and in preterm infants, increases the risk of late-onset sepsis (LOS), necrotizing enterocolitis, retinopathy of prematurity, and death [4].

Different biomarkers are currently used for the diagnosis of neonatal sepsis, such as white blood cell count and absolute neutrophil count, procalcitonin, C-reactive protein, and cytokines, but they have a very low positive predictive value due to the low specificity [5,6,7,8,9]. Blood culture is considered the gold standard for the diagnosis of neonatal sepsis, but its positivity rate is affected by volume of blood submitted for culture, level of bacteremia, or prenatal antibiotic use, which can delay the growth of bacteria and the time to positivity with a negative impact on the sensitivity of the test [10,11].

Recently, new molecular microbiological diagnostic methods that allow shortening the identification time of microorganisms in positive blood cultures have been developed. Hybridization and polymerase chain reaction (PCR) amplification are some of these methods [12,13,14,15]. The FilmArray^®^ Blood Culture Identification (BCID) assay (BioFire Diagnostics, Salt Lake City, UT, USA) has been approved for use in positive blood cultures. However, off-label application of this assay in negative blood cultures is also being evaluated [16,17] in order to reduce the use of antimicrobial therapy. In a pilot study of culture-negative samples from neonates with suspected bloodstream infection, using the BioFire BCID panel at 20 to 24 h of incubation provided 100% true negative results [17].

Following these previous encouraging results [17] and based on the fact that most blood cultures in neonatal bloodstream infections flagged positive during the first 12 h of life [18], a study was designed to assess whether the use of the FilmArray^®^ BCID panel before 20 h continues to maintain a high negative predictive value. In this case, the clinical utility of this assay would contribute not only to reduce the use of empirical antimicrobial treatment but also to prevent initiation of antimicrobial therapy in neonates with suspected early-onset (EOS) and late-onset sepsis (LOS).

## 2. Materials and Methods

### 2.1. Study Design and Participants

A single-center prospective study was conducted in all consecutive neonates with suspected bloodstream infection admitted to the Unit of Neonatology of a tertiary care hospital in Oviedo (Asturias, Spain) between April 2018 and April 2020. Participants were preterm or term neonates with suspicion of neonatal sepsis on clinical grounds or according to the presence of antenatal or perinatal risk factors for infection. Neonatal sepsis was divided into EOS and LOS based on the age of presentation after birth using 72 h as the cutoff. The objective of the study was to assess the diagnostic accuracy of the FilmArray^®^ BCID panel applied to negative blood cultures as compared with final blood culture results obtained after 5 days of incubation. It was hypothesized that availability of results of a rapid test with a high diagnostic accuracy would be clinically relevant to reduce the use of empirical antimicrobial treatment or even to prevent starting antimicrobials in neonates with suspicion of EOS or LOS. The study period corresponded to the first 2 years of implementation of the FilmArray^®^ technique in our institution.

The study protocol was approved by the Clinical Research Ethics Committee of Central University Hospital of Asturias (Oviedo, Spain) (code 2020.285, approval 15 July 2020). The Ethics Committee waived the requirement to obtain the informed consent of parents or legal guardians of neonates because blood sampling was performed in routine conditions of daily practice.

### 2.2. Blood Culture Ascertainment and FilmArray^®^ Assay

Once a neonate presented clinical signs and symptoms of sepsis (such as irritability, tachypnea, respiratory distress, poor feeding, hypotension, hemodynamic instability, etc.) or was identified as requiring sepsis evaluation due to risk factors for sepsis, a blood culture was obtained. A blood sample of 1 mL was drawn and inoculated into a 30 mL blood culture bottle. Then, 0.2 mL were extracted, which was the blood volume required for the FilmArray^®^ assay according to specifications of the manufacturer. The FilmArray^®^ assay does not include specific timing recommendations. The FilmArray^®^ BCID panel (bioMérieux España, Madrid, Spain) is a PCR multiplex system certified by the Food and Drug Administration (FDA), CE-IVD (European Union In Vitro Diagnostic Medical Regulation [IVDR]), and Therapeutics Good Administration (TGA) that analyzes a list of 24 pathogens and 3 genes of antimicrobial resistance (Table 1). Among the microorganisms included in the panel are those involved as causative pathogens in the majority of EOS and LOS diagnosed in neonates in our country.

The FilmArray^®^ BCID panel is approved for use on positive blood cultures for a rapid etiological diagnosis and adjustment of antimicrobial treatment. In the present study, however, the use of the FilmArray^®^ BCID panel was off-label in cultures reported negative for at least 6 h of incubation. All blood cultures were negative at the time of applying the PCR multiplex system. Sepsis was definitively excluded in all neonates in whom a final negative blood culture was obtained.

### 2.3. Study Variables

For each patient the following data were recorded: gestational age; birth weight; risk factors for EOS (such as intrapartum maternal fever, maternal and/or fetal tachycardia, maternal leukocytosis, duration of rupture of membranes, characteristics of the amniotic fluid, maternal antimicrobial therapy, and result of maternal vaginal swab); plasma levels of acute reactants, including interleukin (IL) IL-6 and C-reactive protein (CRP) as routine laboratory analyses when an infection is suspected; microbiological results (peripheral exudates, cerebrospinal fluid, and blood culture); results of the FilmArray^®^ BCID panel and time of incubation; age at diagnosis of sepsis; neonatal clinical manifestations; length of stay; days on antimicrobial treatment; and changes in the management of neonates according to results of the FilmArray^®^ assay. Cutoff values of acute reactants were IL-6 > 300 pg/mL in the first 3 days of life; IL-6 > 85 pg/mL in neonates 3 days old or older; and CRP > 2 mg/dL at any age. These cutpoints, however, were not the only criteria for starting antimicrobial treatment because the neonate’s clinical condition was considered above all.

### 2.4. Statistical Analysis

Categorical variables are expressed as frequencies and percentages, and continuous variables were expressed as mean and standard deviation (SD) or median and interquartile range (IQR). A true positive result was defined when positivity of the FilmArray^®^ assay and the blood culture coincided for the same pathogen; true negative was defined when both techniques were negative; false positive was defined when the FilmArray^®^ was positive, but the blood culture was subsequently negative; and false negative was defined when the FilmArray^®^ assay was negative, but the blood culture was subsequently positive. Descriptive statistics of the study population was performed, and diagnostic accuracy of the FilmArray^®^ BCID panel included the sensitivity, specificity, and positive and negative predictive values with the 95% confidence interval (CI). The SPSS version 24.0 (IBM Corp., Armonk, NY, USA) was used for data analysis.

## 3. Results

### 3.1. Clinical Characteristics of Neonates

The study sample included 102 blood culture specimens recovered from 92 neonates with suspicion of neonatal sepsis admitted to the Unit of Neonatology during the study period. A total of 69 blood cultures were taken from neonates with suspected EOS, and 33 were taken from neonates with suspected LOS. The clinical characteristics of neonates and results of laboratory tests in the groups of EOS and LOS are shown in Table 2. In the group of EOS, 69.6% of neonates had suggestive clinical manifestations of sepsis (such as respiratory distress, tachypnea, fever, vomiting, poor general condition, etc.) and 30.4% were asymptomatic but presented perinatal risk factors for infection (19 cases with suspicion of chorioamnionitis due to intrapartum maternal fever and two cases due to spontaneous preterm delivery at 25 weeks of gestational age). In the group of LOS, there were six asymptomatic neonates in whom blood cultures were indicated because of an increase in biomarkers of bloodstream infection (IL-6 and/or CRP) during routine laboratory analyses.

### 3.2. Diagnostic Accuracy of the FilmArray^®^ BCID Panel

Of the total 102 blood cultures, positivity was detected in 12 cases (11.8%), four of which were in EOS (*Granulicatella adiacens*, *Escherichia coli*, *Listeria*, and coagulase-negative *Staphylococcus* [CoNS], one case each), and eight were in LOS (CoNS in four cases, *E. coli* in one, *Staphylococcus aureus* in two, and *S. agalactiae* in one).

The FilmArray^®^ assay was performed after a median incubation time of the blood cultures of 10 h (IQR 8–20), with 9 h (IQR 8–25) in the EOS group and 10 h (IQR 8–15.7) in the LOS group. Positive results were obtained in eight cases, and negative results were obtained in 94 (Table 3).

The diagnostic accuracy of the FilmArray^®^ BCID panel for the diagnosis of neonatal sepsis showed a 66.7% sensitivity, 100% specificity, 100% positive predictive value, and 95.7% negative predictive value (Table 4). In the group of EOS, the FilmArray^®^ assay showed a 75% sensitivity, 100% specificity, 100% positive predictive value, and 98% negative predictive value, whereas in the group of LOS, there was a 62% sensitivity, 100% specificity, 100% positive predictive value, and 89% negative predictive value.

There were four cases of false negative results; one was in the group of EOS, and three were in the group of LOS. In the false negative case of the EOS group, the causative pathogen was *Granulicatella adiacens*, a Gram-positive coccus not included in the FilmArray^®^ panel. In the three false negative cases detected in the group of LOS, the causative pathogen was *S. epidermidis* in all three, with two cases in the same patient with blood culture incubation periods of 7 and 9 h, respectively, and with 24 h in the remaining patient. Both patients were preterm neonates of less than 28 weeks’ gestation.

The use of the FilmArray^®^ BCID panel allowed the making of a change in the management of 51.9% of neonates with suspicion of sepsis (53 cases of 102 blood cultures), increasing up to 60.8% in the EOS group (42 cases of 69 blood cultures). Of the 53 cases in which a change was made based on negative results of the FilmArray^®^ assay, antimicrobial therapy was not started in 32 cases (60.4%), whereas in the remaining 21 cases (39.6%) it was possible to withdraw the antimicrobial treatment without risks for the neonate. The median length of antimicrobial therapy in blood culture negative cases was 2 days.

## 4. Discussion

In our country and since 1998, various scientific societies have jointly developed a protocol for the prevention of group B *Streptococcus* (GBS) infection in neonates at risk of EOS [5], which includes different approaches from serial clinical assessments to laboratory analysis of infection biomarkers and blood culture. A number of factors, however, have shown an influence on the variability of diagnostic alternatives in the management of newborns at risk, including overdiagnosis of chorioamnionitis (frequently based solely on intrapartum maternal fever), intrapartum antimicrobial therapy that contributes to negative blood cultures, the low specificity and negative predictive value of infection biomarkers used in neonates (IL-6, CRP, procalcitonin), or the lack of specificity of clinical manifestation of sepsis, particularly in preterm newborns [2,6,8]. All these circumstances contribute to overuse of antimicrobials and associated detrimental effects, such as an increase in antimicrobial resistance, emergence of multi-resistant organisms, increased risk of *Candida* spp. colonization and subsequent candidiasis, disruption of the developing neonatal gut microbiome, and higher health care costs [4,19,20,21]. In a systematic review and meta-analysis of the relationship between antibiotic use in neonates and early adverse outcomes, prolonged antibiotic exposure in uninfected infants was associated with an increased risk of necrotizing enterocolitis, invasive fungal infections, and/or death [22].

Although antimicrobials are a central part of the first-line treatment of neonatal sepsis and it is crucial to obtaining an accurate diagnosis and to identify culture-proven sepsis, many challenges remain. Blood culture, the current gold standard for diagnosis of sepsis, suffers from low sensitivity and a reporting delay of approximately 48–72 h, so rapid and sensitive tests that can inform clinicians regarding the institution or optimization of antimicrobial therapy are urgently needed. The ideal diagnostic test should have adequate diagnostic accuracy (specificity and negative predictive value) to reliable exclude sepsis and avoid unnecessary antimicrobial therapy. Molecular microbiological assays offer rapid diagnosis and distinct advantages over blood cultures as they are not influenced by the volume of sample or concurrent antimicrobials use. The FilmArray^®^ assay BCID panel has a favorable diagnostic profile as includes the most common causative pathogens of neonatal EOS and LOS in our environment [19]. On the other hand, in cases of early-onset sepsis (EOS), negative blood cultures may be associated with transplacental passage of antimicrobials when intrapartum antimicrobials are administered to the mother. This is a well-known limitation of blood culture in this clinical setting. The use of the FilmArray^®^ assay may reduce false negative results related to maternal intrapartum antimicrobial treatment given that the FilmArray^®^ technique is based on analysis of bacterial DNA in blood and does not require the presence of living pathogens in the sample.

In the present study, the FilmArray^®^ assay was used in blood negative cultures (off- label) to assess the negative predictive value of the test in order to avoid or reduce the use of empirical antimicrobial therapy in neonates with suspected sepsis. Similarly, a previous study of Massa-Buck et al. [17] has demonstrated successful off-label application of the FilmArray^®^ assay to identify microorganisms from neonatal blood cultures with suspected sepsis prior to positivity. We found a 100% specificity, 95.7% negative predictive value, and four cases of false negative results. In one case, *Granuticatella adiacens* was isolated, a pathogen not included in the FilmArray^®^ panel and an exceptional etiology of EOS [19,23]. The remaining three cases were caused by CoNS in neonates with LOS, and in two of them the incubation period until performance of the molecular test was less than 10 h. This incubation period and/or the fact that it may be contaminants (low concentration of the microorganism) rather than true sepsis (usual finding in CoNS isolates in neonatal units) may be the reason for negative results of the FilmArray^®^ assay [24].

However, from a clinical perspective, the negative predictive value of the test allowed empirical antimicrobial therapy to be reduced or even never used in 51.9% of neonates with suspected sepsis (clinical manifestations and/or elevated biomarkers), which represents a considerable reduction in the use of antimicrobials. In normal conditions, the expected duration of antimicrobial treatment would be around 5 days, when the final report of the blood culture is usually provided. Based on the present findings, the FilmArray^®^ assay BCID panel is currently an essential tool for guiding antimicrobial decision making in neonates with suspected sepsis admitted to our unit.

The single-center design and the small number of blood culture specimens evaluated are limitations of the study. Finally, we have no experience with the use of 16S rRNA PCR testing or MALDI-TOF mass spectrometry, but the effectiveness of these techniques for a rapid diagnosis of neonatal sepsis especially in blood culture-negative cases merits further evaluation.

## 5. Conclusions

In the present study, the use of the FilmArray^®^ BCID panel in negative blood cultures prior to 20 h of incubation showed a high specificity and negative predictive value, which makes this test a useful tool in decision-making on starting or early withdrawal of empirical antimicrobial treatment in neonatal sepsis.

## Figures and Tables

**Table 1:** FilmArray^®^ blood culture identification panel (BCID).

Gram-positive bacteria	*Enterococcus*
	*Listeria monocytogenes*
	*Staphylococcus*
	*Staphylococcus aureus*
	*Streptococcus*
	*Streptococcus agalactiae*
	*Streptococcus pyogenes*
	*Streptococcus pneumoniae*
Gram-negative bacteria	*Acinetobacter baumannii*
	*Haemophilus influenzae*
	*Neisseria meningitidis*
	*Pseudomonas aeruginosa*
	*Enterobacteriaceae*
	*Enterobacter cloacae complex*
	*Escherichia coli*
	*Klebsiella oxytoca*
	*Klebsiella pneumoniae*
	*Proteus*
	*Serratia marcescens*
Yeasts	*Candida albicans*
	*Candida glabrata*
	*Candida krusei*
	*Candida parapsilosis*
	*Candida tropicalis*
Antimicrobial resistance genes	mecA: methicillin resistance
	Van-A/Van-B: vancomycin resistance
	KPC: carbapenem-resistant *K.pneumoniae*

**Table 2:** Clinical and laboratory data in infants with suspected neonatal sepsis.

Study Variables	Early-Onset Sepsis (*n* = 69)	Late-Onset Sepsis (*n* = 33)
Gestational age, weeks, mean (SD)	37.8 (3.7)	31.8 (5.7)
Birth weight, g, mean (SD)	2934 (738)	1852 (1109)
Antenatal risk factors *		
Maternal vaginal swab for group B *Streptococcus*, *n* (%)		
Negative	46 (66.7)	
Positive	11 (15.9)	
Unknown	12 (17.4)	
Maternal antimicrobial prophylaxis, *n* (%)		
Yes	26 (37.7)	
No	41 (54.9)	
Unknown	2 (2.9)	
Time of membrane rupture, hours, median (IQR)	11.0 (5–24)	
Intrapartum maternal fever (≥38 °C), *n* (%)		
Yes	39 (56.5)	
No	24 (34.8)	
Unknown	6 (8.7)	
Asymptomatic at diagnosis, *n* (%)	21 (30.4%)	6 (18.2)
Clinical signs and symptoms at diagnosis, *n* (%)	48 (69.6)	27 (81.8)
Acute phase reactants		
IL-6, pg/mL		
Median (IQR)	446.5 (80–1330)	145 (47–300)
≥300 pg/mL, *n* (%)	36 (52.2)	
≥85 pg/mL, *n* (%)		21 (63.6)
C-reactive protein, mg/dL		
Median (IQR)	2.1 (0.2–4.3)	3.5 (1.7–9.2)
≥2 mg/dL, *n* (%)	35 (50.7)	22 (66.7)

* Early-onset neonatal sepsis only.

**Table 3:** Positive and negative results of blood cultures and the FilmArray^®^ BCID panel in 102 samples.

FilmArray^®^ Assay	Blood Culture		Total
	Positive	Negative	
**Positive**	8	0	8
**Negative**	4	90	94
**Total**	12	90	102

**Table 4:** Accuracy of the FilmArray^®^ BCID panel for the diagnosis of neonatal sepsis compared with blood culture.

FilmArray^®^ Assay	All Neonatal Sepsis	Early-Onset Sepsis	Late-Onset Sepsis
Diagnostic accuracy, % (95% CI)			
Sensitivity	66.7 (32–100)	75 (20–100)	62 (19–100)
Specificity	100	100	100
Positive predictive value	100	100	100
Negative predictive value	95.7 (90–100)	98 (94–100)	89 (74–100)

CI: confidence interval.

## Data Availability

Data of the study are available from the corresponding author upon request.
